# Severe Maternal Morbidity and Mortality After Delivery Hospitalization Among Rural Residents Bypassing Local Care for Urban Hospitals

**DOI:** 10.1001/jamanetworkopen.2025.44522

**Published:** 2025-11-19

**Authors:** Peiyin Hung, Haoyuan Gao, Jihong Liu, A. Caroline Rudisill, Nansi S. Boghossian, Berry A. Campbell, Lauren Workman, Yunqing Ma, Jiajia Zhang

**Affiliations:** 1 University of South Carolina Rural Health Research Center, Columbia; 2 Department of Health Services Policy & Management, Arnold School of Public Health, University of South Carolina, Columbia; 3 South Carolina SmartState Center for Healthcare Quality, University of South Carolina, Columbia; 4 Department of Epidemiology and Biostatistics, Arnold School of Public Health, University of South Carolina, Columbia; 5 Department of Health Promotion, Education, and Behavior, Arnold School of Public Health, University of South Carolina, Columbia; 6 Division of Maternal-Fetal Medicine, University of South Carolina School of Medicine, Columbia; 7 Center for Applied Research and Evaluation, University of South Carolina, Columbia

## Abstract

**Importance:**

Rural residents experience higher severe maternal morbidity and mortality (SMMM) and limited local hospital obstetric supply. However, data on postpartum SMMM among these rural residents bypassing local communities for childbirth are limited.

**Objective:**

To estimate postpartum SMMM among urban, rural nonlocal, and rural local births.

**Design, Setting, and Participants:**

This retrospective population-based cohort study identified all childbirth deliveries and hospitalization discharges from January 1, 2018, to December 31, 2022, in South Carolina and followed-up to 1-year postpartum using data from all-payer hospital inpatient, outpatient, and emergency department visits linking to vital records for birth and death certificates information. Data were analyzed from March to September 2025.

**Exposure:**

Urban residency, rural residency with nonlocal birth (ie, urban hospital deliveries), and rural residency with local birth (deliveries at in-county or adjacent rural hospitals), classified using the 2023 Rural-Urban Continuum Codes definitions (1-3, urban; 4-9, rural).

**Main Outcome and Measures:**

The primary outcomes of postpartum SMMM—severe maternal morbidity (SMM) and/or pregnancy-associated mortality—were identified using *International Statistical Classification of Diseases and Related Health Problems, Tenth Revision* diagnosis and procedure codes and death certificates, respectively. Cox proportional hazards models compared cumulative incidence and timing of postpartum SMMM across exposure groups.

**Results:**

Of 235 375 deliveries to 197 216 women (mean [SD] maternal age, 28.3 [5.8] years), 203 325 (86.4%) were to urban residents, 15 053 (6.4%) were to rural residents bypassing local birth sites for urban hospitals, and 16 997 (7.2%) were to rural residents delivering locally, yielding a 47.0% bypassing rate for rural residents. SMMM was highest among rural nonlocal deliveries (180.0 per 10 000 births), followed by similar rates for urban (118.8 per 10 000 births) and rural local deliveries (114.7 per 10 000 births). Adjustment for maternal sociodemographic characteristics, clinical factors, and hospital characteristics still showed higher SMMM risk for rural nonlocal vs urban deliveries (adjusted hazard ratio, 1.18; 95% CI, 1.04-1.33), while the risk associated with rural local vs urban delivery was not significantly different.

**Conclusions and Relevance:**

In this cohort study of 2018 to 2022 childbirth deliveries in South Carolina, rural local deliveries had SMMM risks comparable to urban births, but rural nonlocal deliveries were associated with increased risk. These findings suggest that targeted interventions (eg, strengthening rural obstetric care supply, childbirth discharge planning, postpartum care coordination, and timely follow-up) may help mitigate these disparities for rural nonlocal births.

## Key Points

**Question:**

Do rural residents who bypassed local communities for delivery at urban hospitals (nonlocal delivery) have higher postpartum severe maternal morbidity and mortality (SMMM) than rural local and urban births?

**Findings:**

In this cohort study of 235 375 births in South Carolina, nearly one-half of rural deliveries occurred at nonlocal hospitals. Rural nonlocal deliveries had the highest 1-year postpartum SMMM risk, even after adjusting for maternal and hospital factors.

**Meaning:**

In this cohort study, rural nonlocal births were more likely to experience SMMM than urban and rural local births, underscoring the need for targeted discharge planning and postpartum care coordination for rural deliveries in nonlocal settings.

## Teaser

This cohort study compares risk of severe maternal morbidity and mortality among women in South Carolina who bypassed local communities for delivery at urban hospitals (nonlocal delivery) vs a local rural or urban hospital.

## Introduction

Severe maternal morbidity (SMM)—potentially life-threatening conditions that manifest during pregnancy, at delivery, or postpartum—results in significant short- or long-term consequences for a woman’s health.^1^ These complications are frequently associated with long hospital stays and high medical costs, and if not properly treated, could result in maternal death.^2,3,4^ Annually, approximately 60 000 individuals nationwide experience SMM, a critical near miss from pregnancy-associated deaths, which claim the lives of 700 individuals every year in the US.^5^ SMM and pregnancy-associated mortality (SMMM) disproportionately affect rural populations,^6,7,8,9,10^ with the maternal mortality ratio for rural residents almost 2-fold that of urban residents.^11^ In South Carolina—a southern rural state with the eighth highest pregnancy-associated death rate in the nation—maternal mortality in rural counties (55.7 deaths per 100 000 live births) is nearly twice as high as in urban areas (28.9 deaths per 100 000 live births).^12^ More than 70% of these deaths occurred during the postpartum period.^12^

Access to quality maternity care and social determinants of health (eg, travel burdens or care access) are key factors underlying rural disparities.^13,14,15,16,17^ However, access to care has been decreasing nationwide. From 2004 to 2018, rural America experienced a 19% reduction in hospital-based obstetric units, much higher than the 5% reduction observed in urban areas.^16,18,19^ In South Carolina alone, 13 hospital obstetric units have closed since 2012, and as of 2022, more than one-half of South Carolina counties were designated as medically underserved areas. These closures have hindered access to timely and adequate perinatal care.^12,20^

Historically, many rural residents have bypassed local facilities to seek high-acuity obstetric care in urban hospitals due to clinical needs.^21,22^ Recent closures have further exacerbated this trend, increasing travel burdens and widening existing maternal health disparities in these vulnerable populations.^19,23^ However, data on SMMM among these rural residents bypassing local hospitals for childbirth are limited. This study used all-payer data linking to vital records to estimate SMMM among urban, rural nonlocal, and rural local births. This is the first study of which we are aware to use such all-payer outpatient, inpatient, and emergency department data to build an encompassing picture of postdischarge SMMM risk and examine how place of birth relative to home is associated with SMMM risk. We hypothesized SMMM risk to be greatest among rural nonlocal births, moderate among rural local births, and lowest among urban births.

## Methods

### Study Design and Data

This retrospective population-based cohort study was approved by the University of South Carolina institutional review board and followed the Strengthening the Reporting of Observational Studies in Epidemiology (STROBE) reporting guideline. We extracted data from the South Carolina all-payer hospital inpatient, outpatient, and emergency department utilization database, linking it to vital records birth and death certificate registries from 2018 to 2023. We first extracted childbirth hospitalizations and stillbirths using previously documented *International Statistical Classification of Diseases and Related Health Problems, Tenth Revision (ICD-10)* diagnosis or procedure codes, or diagnostic related group codes. We then linked each hospitalization record to birth certificates records using unique patient identifiers and labor and delivery dates. Death registry records were used to flag pregnancy-associated mortality for a patient from childbirth to 1-year postpartum.

### Study Cohort

The study cohort included all 235 375 childbirths from individuals aged 15 to 50 years who gave birth in South Carolina, from January 1, 2018, to December 31, 2022, regardless of payer status (eFigure 1 in Supplement 1). Multiple deliveries from a single patient were included when they were separated by at least 160 days. Of 243 317 deliveries to 203 323 birthing individuals from the linkage of hospital-based childbirth records and birth certificate data, 7942 births to 6109 birthing individuals were excluded due to missing information on maternal age, race and ethnicity, residential county, education, prenatal care pattern, plurality, and parity. Follow-up data extended through December 31, 2023, allowing up to 1 year of postpartum observation for the entire cohort.

### Measures

The primary outcome was days from discharge to the first episode of SMM or pregnancy-associated mortality (postpartum SMMM)^24,25,26^ or to 365 days postdischarge for censored individuals without an event). We chose the postpartum timing of SMMM because it marks a critical transition time from hospital to residential community settings, and this approach allows everyone to be consistently followed up for 365 days from discharge. SMM was identified from hospital inpatient, outpatient, and emergency department utilization records, using *ICD-10* diagnosis and procedure codes for 20 conditions (eg, eclampsia, sepsis, disseminated intravascular coagulation, acute myocardial infarction, or acute respiratory distress) compiled by the Centers for Disease Control and Prevention. Because blood transfusion alone may not represent a truly severe maternal event,^27^ such cases were not considered as SMM. Mortality was defined using death certificates data with all-cause deaths that occurred during childbirth or within 1 year postdischarge. Rates of SMMM were calculated as the number of deliveries that experienced SMMM per 10 000 births.

Residential and childbirth hospital locations were used to categorize participants into 3 exposure groups: (1) urban residents, (2) rural residents who gave birth in nonlocal hospitals, and (3) rural residents who gave birth in local hospitals. We did not categorize urban residents by their birthing location because only about 5% gave birth in nonlocal settings (ie, rural, nonadjacent counties) (eFigure 2 in Supplement 1). Among rural residents, we distinguished those who bypassed rural hospitals for childbirth in urban facilities (nonlocal births) from those who delivered at in-county or adjacent-county rural hospitals (local births). We defined urban (Rural-Urban Continuum Codes [RUCCs] 1-3) or rural location (RUCCs 4-9), based on residence county, using the 2023 RUCC definitions from the US Department of Agriculture.^28^

We extracted delivery- and hospital-level covariates that were evidently associated with adverse maternal outcomes and care settings.^26,29,30^ Delivery-level covariates included maternal age at birth, race and ethnicity, education attainment, parity, Kotelchuck Prenatal Care Adequacy Index, gestational age at birth, obstetric comorbidity index, mode of delivery, and birth year. Race and ethnicity were self-reported by parents in their child’s or children’s birth certificates. Race and ethnicity categories included Hispanic, non-Hispanic Black, non-Hispanic White, and non-Hispanic other race or ethnicity (ie, American Indian and Alaska Native, Asian, multiracial, and Native Hawaiian or Other Pacific Islander); race and ethnicity were included to account for different care experiences across groups. Obstetric Comorbidity Index summary score was calculated using all-payer hospital data from pregnancy conception to childbirth discharge based on a previously published algorithm^31^ (excluding preterm birth indicator to separately adjust for gestational age at birth), ranging from 0 to 118, with higher scores indicating higher risk of SMMM. Birth month was further categorized based on 1-year postpartum exposure to the COVID-19 public health emergency (PHE): those discharged before March 1, 2019 (unexposed), and those discharged on or after March 1, 2020 (exposed).

Hospital-level covariates (hospital obstetric care level [Level I, II, and III-IV]) and hospital obstetric care workforce model [no obstetricians, obstetrician only, and both obstetrician and family physicians]) were constructed using all-payer hospital data for the childbirth hospitalization record. A physician who had taxonomy of obstetrics and other specialties (eg, family medicine) was considered as an obstetrician.

### Statistical Analysis

The association of delivery with hospital characteristics of childbirths by residence and clinician location were first examined using χ^2^ tests. Second, Kaplan-Meier estimates were used to determine cumulative incidence of SMMM at predefined time intervals postpartum. Cumulative incidence of SMMM during follow-up from childbirth hospital discharge to the first event was compared by residence and childbirth location using log-rank tests. Third, we generated unadjusted rates of SMM per 10 000 births by location. Fourth, we conducted multivariable Cox proportional hazard models, adjusting for aforementioned covariates. Birth year fixed effects were used to control for secular trends. Several sensitivity analyses were conducted: by postpartum exposure to COVID-19 PHE, across prenatal care receipt at the same vs different site as childbirth delivery hospital or no prenatal care, and the differential associations of locations with SMMM by maternal race and ethnicity. Tests of the associations between time and Schoenfeld residuals showed no violations for the proportional hazards assumption across all models. Statistical significance was determined at a 2-sided *P* < .05. Analyses were performed from March to September 2025 using R version 4.3.2 (R Project for Statistical Computing).

## Results

### Study Sample

**Figure 1.**
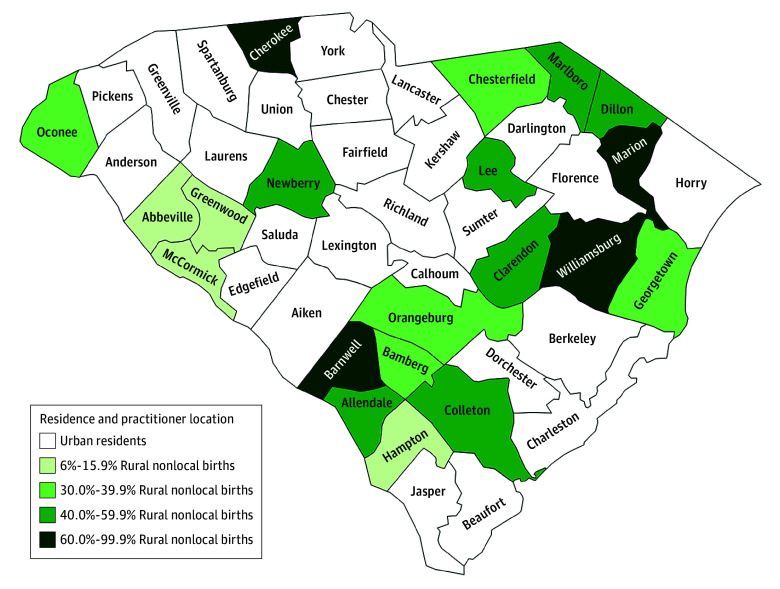
County Distributions of Urban Births and Proportion of Rural Nonlocal Births Among Rural Counties in 2018-2022 Urban (rural-urban continuum codes [RUCCs] 1-3) or rural location (RUCCs 4-9), based on residence county, was defined using the 2023 RUCCs developed by the US Department of Agriculture.

Among 235 375 deliveries from 2018 to 2022 to 197 216 individuals (mean [SD] maternal age, 28.3 [5.8] years; 12 813 Hispanic [5.44%], 71 473 non-Hispanic Black [30.4%], and 134 925 non-Hispanic White individuals [52.3%]), 203 325 (86.4%) were to urban residents, 15 053 (6.4%) were to rural residents who bypassed local hospitals for urban childbirth (rural nonlocal deliveries), and 16 997 (7.2%) were to rural residents who delivered locally (rural local deliveries) (Table 1). Of rural deliveries alone, nearly one-half (15 053 [47.0%]) were nonlocal, with substantial variations from 223 of 3538 deliveries (6.3%) in Greenwood County, South Carolina to 2780 of 2782 deliveries (99.9%) in Cherokee County, South Carolina (Figure 1). Compared with urban residents, rural nonlocal deliveries were by mothers who were younger, identified as non-Hispanic Black, had lower educational attainment, and who were more likely to undergo cesarean delivery. Rural nonlocal deliveries were more likely to be preterm and by mothers who had higher rates of obesity, who were more likely to receive inadequate prenatal care, and who had a higher comorbidity score. These rural nonlocal deliveries were more likely to be in hospitals with higher obstetric care level and with both obstetricians and family physicians attending births.

**Table 1.** Maternal and Hospital Characteristics by Residence and Childbirth Hospital Location

Characteristic	All study childbirths, No. (%) (N = 235 375)	Residents by hospital location, No. (%)^a^		
		Urban (n = 203 325)	Rural	
			Nonlocal birth (n = 15 053)	Local birth (n = 16 997)
Maternal age at birth, y				
<20	12 767 (5.4)	10 299 (5.1)	947 (6.3)	1521 (9.0)
20-24	51 278 (21.8)	42 071 (20.7)	4144 (27.5)	5063 (29.8)
25-29	71 593 (30.4)	61 528 (30.3)	4872 (32.4)	5193 (30.6)
30-34	63 516 (27.0)	56 771 (27.9)	3245 (21.6)	3500 (20.6)
≥35	36 221 (15.4)	32 656 (16.1)	1845 (12.3)	1720 (10.1)
Maternal race and ethnicity				
Hispanic	12 813 (5.4)	11 779 (5.8)	177 (1.2)	857 (5.0)
Non-Hispanic Black	71 473 (30.4)	57 682 (28.4)	5929 (39.4)	7862 (46.3)
Non-Hispanic White	134 925 (57.3)	118 980 (58.5)	8320 (55.3)	7625 (44.9)
Non-Hispanic other^b^	16 164 (6.9)	14 884 (7.3)	627 (4.2)	653 (3.8)
Maternal education attainment				
No high school diploma	28 746 (12.2)	23 997 (11.8)	1750 (11.6)	2999 (17.6)
High school diploma	61 292 (26.0)	50 152 (24.7)	4882 (32.4)	6258 (36.8)
Some college	76 654 (32.6)	65 404 (32.2)	5665 (37.6)	5585 (32.9)
Bachelor’s degree	43 841 (18.6)	40 513 (19.9)	1845 (12.3)	1483 (8.7)
Graduate school	24 842 (10.6)	23 259 (11.4)	911 (6.1)	672 (4.0)
Parity				
1	87 616 (37.2)	76 334 (37.5)	5491 (36.5)	5791 (34.1)
2	74 685 (31.7)	64 796 (31.9)	4731 (31.3)	5158 (30.4)
3	42 491 (18.1)	36 491 (18.0)	2715 (18.0)	3285 (19.3)
≥4	30 583 (13.0)	25 704 (12.6)	2116 (14.1)	2763 (16.3)
Gestational age at birth, wk				
≤31	3521 (1.5)	2904 (1.4)	473 (3.1)	144 (0.9)
32-33	2825 (1.2)	2413 (1.2)	312 (2.1)	100 (0.6)
34-36	18 447 (7.8)	15 704 (7.7)	1558 (10.4)	1185 (7.0)
37-38	71 412 (30.3)	61 151 (30.1)	4875 (32.4)	5386 (31.7)
39-40	132 972 (56.5)	115 669 (56.9)	7593 (50.44)	9710 (57.13)
≥41	6198 (2.6)	5484 (2.7)	242 (1.6)	472 (2.8)
Kotelchuck Index				
Inadequate	39 435 (16.8)	33 821 (16.6)	2858 (19.0)	2756 (16.2)
Intermediate	12 357 (5.3)	10 727 (5.3)	596 (4.0)	1034 (6.1)
Adequate	67 342 (28.6)	59 351 (29.2)	3565 (23.7)	4426 (26.0)
Adequate plus	116 067 (49.3)	99 285 (48.8)	8017 (53.3)	8765 (51.6)
Obstetric Comorbidity Index				
None	61 879 (26.3)	54 477 (26.8)	2866 (19.0)	4536 (26.7)
1-8	67 132 (28.5)	57 752 (28.4)	4120 (27.4)	5260 (31.0)
9-14	55 825 (23.7)	48 149 (23.7)	3638 (24.2)	4038 (23.8)
≥15	50 539 (21.5)	42 947 (21.1)	4429 (29.4)	3163 (18.6)
Hospital obstetric care level				
I	32 255 (13.7)	25 460 (12.5)	437 (2.9)	6358 (37.4)
II	90 974 (38.7)	79 192 (39.0)	5641 (37.5)	6141 (36.1)
III-IV	90 186 (38.3)	78 393 (38.6)	7476 (49.7)	4317 (25.4)
Missing	21 960 (9.3)	20 280 (10.0)	1499 (10.0)	181 (1.1)
Hospital obstetric care workforce model				
No obstetrician	39 (<0.1)	26 (<0.1)	<10	11 (0.1)
Obstetrician only	82 115 (34.9)	73 237 (36.0)	>3800 (25.8)	4988 (29.4)
Both obstetrician and family physician	153 221 (65.1)	130 062 (64.0)	11 161 (74.1)	11 998 (70.6)
Birth year				
2018	42 928 (18.2)	36 979 (18.2)	2609 (17.3)	3340 (19.7)
2019	44 671 (19.0)	38 526 (19.0)	2738 (18.2)	3407 (20.0)
2020	46 229 (19.6)	39 821 (19.6)	2951 (19.6)	3457 (20.3)
2021	49 043 (20.8)	42 437 (20.9)	3240 (21.5)	3366 (19.8)
2022	52 504 (22.3)	45 562 (22.4)	3515 (23.4)	3427 (20.2)

^a^ *P* values for statistical differences in maternal and hospital characteristics across urban, rural nonlocal, and rural local births groups were all less than .001, calculated using Pearson χ^2^ tests.

^b^ Non-Hispanic other includes American Indian and Alaska Native, Asian, multiracial, and Native Hawaiian or Other Pacific Islander.

### Incidence Rates of SMMM

A total of 2881 deliveries (122.4 per 10 000 births) had SMMM within 365 days postpartum. The median (IQR) time to event was 66 (10-208) days for SMMM-affected deliveries. Rural nonlocal deliveries had the highest rates (180.0 per 10 000 births), followed by urban resident deliveries (118.8 per 10 000 births) and rural local deliveries (114.7 per 10 000 births) (*P* < .001) (Table 2 and eTable 1 in Supplement 1).

**Table 2.** Rates and Risk of Severe Maternal Morbidity and/or Mortality by Residence and Birthing Hospital Location

Characteristic	Severe maternal morbidity and/or mortality cases, No. (rate/10 000 births)	*P* value	Severe maternal morbidity and/or moratlity, HR (95% CI)		*P* value
			Unadjusted	Adjusted^a^	
Severe maternal morbidity and mortality, all^b^	2881 (122.4)^c^	NA	NA	NA	NA
Residence and birthing hospital location					
Urban residents	2415 (118.8)	<.001	1 [Reference]	1 [Reference]	NA
Rural residents bypassed for urban hospitals	271 (180.0)		1.51 (1.33-1.71)^c^	1.18 (1.04-1.33)^c^	.01
Rural residents staying at local hospitals	195 (114.7)		0.97 (0.84-1.12)	0.88 (0.76-1.03)	.11
Severe maternal morbidity, all	2866 (121.8)^c^				
Urban residents	2401 (118.1)	<.001	1 [Reference]	1 [Reference]	
Rural residents bypassed for urban hospitals	269 (178.7)		1.50 (1.32-1.70)^c^	1.17 (1.03-1.33)^c^	.02
Rural residents staying at local hospitals	196 (115.3)		0.98 (0.84-1.13)	0.90 (0.77-1.04)	.16
Pregnancy-associated mortality, all	125 (5.3)^c^				
Urban residents	96 (4.7)	<.001	1 [Reference]	1 [Reference]	NA
Rural residents bypassed for urban hospitals	16 (10.6)		2.28 (1.34-3.86)^c^	1.65 (0.97-2.82)	.07
Rural residents staying at local hospitals	13 (7.7)		1.64 (0.92-2.92)	1.54 (0.84-2.84)	.16

Abbreviations: HR, hazard ratio; NA, not applicable.

^a^ Adjusted HRs were calculated from a multivariable Cox proportional hazard model, controlling for maternal age at birth, race and ethnicity, education attainment, parity, Kotelchuck Prenatal Care Adequacy Index, gestational age at birth, Obstetric Comorbidity Index, hospital obstetric care level, hospital obstetric care workforce model, and birth year. Full model results can be found in eTable 2 in Supplement 1.

^b^ Because blood transfusion alone may not represent a truly severe maternal event, we did not consider such cases as severe maternal morbidity or mortality.

^c^ Significant values.

**Figure 2.**
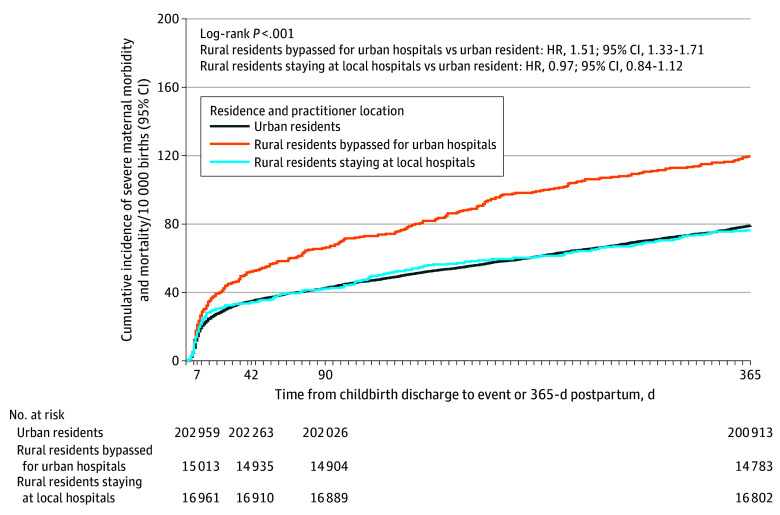
Cumulative Incidence of Severe Maternal Morbidity and Pregnancy-Associated Mortality From Childbirth Discharge to 365 Days Postpartum Hazard ratios (HRs) were calculated from a Cox proportional hazard model. Cumulative incidence of severe maternal morbidity and mortality is shown for deliveries to urban residents, rural residents who bypassed for urban hospitals, and rural residents who stayed locally at rural hospitals from January 1, 2018, December 31, 2023.

Cumulative incidence curves showed the greatest divergence in postpartum SMMM risk among rural nonlocal deliveries, particularly beyond 90 days postpartum (Figure 2). At the 365-day follow-up, compared with urban resident deliveries, rural local deliveries had similar SMMM risk (unadjusted hazard ratio [HR], 0.97; 95% CI, 0.84-1.12), while rural nonlocal births had higher risk (HR, 1.51; 95% CI, 1.33-1.71; *P* < .001) (Figure 2). In the fully adjusted model, rural nonlocal individuals remained at higher risk of SMMM (adjusted hazard ratio [aHR], 1.18; 95% CI, 1.04-1.33; *P* = .01) (Table 2). eTable 2 in Supplement 1 presents the full model results. Results were consistent for SMM alone; for pregnancy-associated mortality, rural nonlocal deliveries had higher, but not statistically significant, risk after adjustment (Table 2).

Before the COVID PHE, rural residents, whether delivering locally or bypassing, had similar SMMM risks compared with urban residents (eTable 3 in Supplement 1). During the COVID PHE, rural individuals with nonlocal births had higher SMMM risk than urban individuals (aHR, 1.22; 95% CI, 1.06-1.41; *P* = .005). In contrast, rural residents who delivered locally did not differ significantly from urban individuals (aHR, 0.83; 95% CI, 0.70-1.06; *P* = .06). Additional analyses suggested that whether one received prenatal care at the childbirth delivery hospital was not significantly associated with postpartum SMMM (eTable 4 in Supplement 1). Racial and ethnic differences in SMMM were persistent regardless of residence and hospital locations (eTable 5 in Supplement 1).

## Discussion

In this population-based cohort study of South Carolina births, we found rural residents who delivered at urban hospitals—accounting for nearly one-half of rural births—experienced the highest risk of SMMM, significantly exceeding the risk for urban residents and rural residents delivering locally. This finding suggests that not only was rural residence associated with higher SMMM risk, but traveling outside one’s local area for childbirth, whether due to clinician referral, patient choice, or other factors, appears to compound that risk.

Our findings of rural-urban disparities in SMMM are consistent with prior literature. A national study from 2007 to 2015 found that rural birthing people, compared with their urban peers,^9^ had elevated risk of SMM and in-hospital mortality by 9% during childbirth hospitalization. This nationwide finding parallels our data of higher SMMM among South Carolina’s rural 2018 to 2022 birthing population, reinforcing the rural-urban gap across states and years. According to the South Carolina maternal mortality review committee, in 2018 to 2021, rural births had 62% higher pregnancy-associated mortality rates than urban births with 55.4 vs 34.2 per 100 000 births.^12^ Our study adds a new nuance to the existing literature by differentiating rural deliveries into local vs nonlocal and found that hospital location plays a critical role in differentiating SMMM risk. The heightened SMMM risk we identified among rural residents, especially those traveling for urban hospitals to give birth during the COVID-19 PHE, is part of a broader pattern of rural maternal health disadvantage documented across the US.^32,33,34^

The fact that rural mothers delivering nonlocally—those likely traveling long distances to give birth—had the greatest SMMM risk suggests that rural risk is not monolithic and where the delivery takes place matters. Travel distance and access to obstetric units have evidently shown to be associated with maternal outcomes. A 2011 to 2015 Pennsylvania study found that patients living 37 to 50 miles away vs 3 or fewer miles away from a birthing hospital had 53% higher SMM risk.^33^ While nationwide research on the association of distance to care with SMM or SMMM is lacking, the recent loss of rural obstetric units has exacerbated such accessibility to care, forcing many rural mothers to give birth far from home.^15,16^ Indeed, fewer than 50% of rural US birthing individuals have access to obstetric units within a 30-minute drive, and more than 10% must drive more than 100 miles for maternity care.^35,36^ South Carolina mirrors this challenge as 100% of rural South Carolina residents live more than 30 minutes from a birthing hospital, compared with only 8.5% of urban residents.^20^ Rural individuals delivering outside their local area did so likely because nearer options were unavailable or lacked appropriate services, contributing to delayed or complicated diagnosis, resulting in higher SMMM.

Our results showed a shift in postpartum SMMM risk during the COVID-19 PHE. Although rural and urban residents had similar risks before the PHE, rural residents who gave birth at urban hospitals had a higher SMMM risk than urban residents. In contrast, rural residents who delivered locally had a modestly lower SMMM risk, findings that carry several key implications. These shifts likely reflect structural changes during COVID-19 PHE, including recent increases in rural obstetric unit closures^19^ and the emergence of maternity care deserts.^37^ Meanwhile, the rapid expansion of telemedicine might have helped individuals delivering locally maintain access to prenatal and postpartum support, buffering risk.^38^ Persistent structural barriers, such as broadband gaps, limited telecommunication resources, and heightened stress and anxiety during the PHE, likely compounded risks for rural residents traveling to urban hospitals for obstetric care.^17,39,40^ Future studies should disentangle the roles of structural factors and psychosocial stressors to inform policies aimed at reducing maternal health disparities in rural communities.

The increased risk of postpartum SMMM among rural nonlocal births highlights the need for targeted interventions, such as expanding rural obstetric care supply in high-risk communities, and/or facilitating care coordination during childbirth hospitalization, comprehensive discharge planning, and robust postpartum follow-up in urban obstetric care settings delivering rural births. Given that more than 70% of pregnancy-associated mortality occurred after hospital discharge,^12^ and that rural hospital closures and workforce shortages have increased rural non-local births,^15,16,18,21^ our findings highlight the need to address the potential consequences of rural obstetric bypassing. First, urban clinicians should recognize rural patients who travel for childbirth to their settings as high risk and treat proactively to mitigate SMMM risk during the immediate and extended postpartum periods. The Hear Her Campaign has recognized the urgent maternal warning signs of pregnancy complications, providing an opportunity to leverage this by developing standardized protocols for inpatient education on warning signs of common postpartum complications^41,42,43^; this is particularly important for rural patients delivering nonlocally because our data indicate that their educational attainment is relatively low. Second, similar to psychiatric hospital discharge planning,^44,45^ interhospital coordination and care transition might help ensure rural birthing individuals have prearranged appointments with a local perinatal practitioner as part of discharge plan. Third, leveraging current community-based home visiting programs to provide informational, instrumental, and social support might help address barriers to care continuity between hospital childbirth care and community postpartum care postdischarge.^46,47^

### Limitations

There are limitations to consider. First, the retrospective design and administrative data hinder our abilities to conclude causality. Second, we defined bypass and nonlocal based on a patient’s county relative to the hospital county, which may not fully capture actual proximity to childbirth due to data limitations. Therefore, some high-acuity rural births that require travel to adjacent counties may be classified as local, potentially underestimating the bypassing association with SMMM; this is especially true if travel distance and adverse maternal outcomes are positively associated.^33^ Also, the higher risks observed among rural residents who received care at urban centers may reflect unobservable factors such as other underlying medical risk factors such as fetal conditions, health literacy, quality of care, or proximity to the nearest obstetric care and postpartum providers. However, efforts were made to control for clinical reasons that could explain bypassing for urban hospitals, such as gestational age at birth, plurality, and obstetric comorbidity index which incorporates 26 comorbidities in relation to SMM.^31^ Third, while our findings align with national rural-urban SMMM gaps, our results may not apply to other states, depending on health care infrastructure, Medicaid expansion, patient mix, and rural hospital obstetric capacity. Fourth, our SMM identification relied on hospital-based records, which might be underestimated by missing complications treated in nonhospital settings. Also, our focus on postpartum SMM ensured consistent 1-year follow-up across all deliveries but did not capture intrapartum SMM that might have been present before the delivery and/or a result of antepartum complications rather than outcomes following delivery, which warrant future investigations. Moreover, while we examined postpartum SMMM up to 1-year postdischarge, we could not fully distinguish between preventable vs unavoidable complications leading to readmission. More detailed cause-specific analyses (eg, postpartum hemorrhage vs cardiomyopathy-related admissions) could provide further evidence into targetable risk factors. Despite these limitations, to our knowledge, this is the first study measuring postpartum SMMM from all-payer outpatient, inpatient, and emergency department records, linking to death certificates records. The results provide compelling evidence on persistent SMMM disparities facing rural populations and highlight critical areas for intervention.

## Conclusions

In this cohort study of 2018 to 2022 childbirth deliveries in South Carolina, rural local births had SMMM risks comparable to urban births, but bypassing local hospitals was associated with increased SMMM risk. Targeted interventions that facilitate childbirth discharge planning, postpartum care coordination, and timely follow-up may help mitigate these disparities for rural nonlocal births. Ongoing efforts should focus on keeping rural birthing individuals engaged in care after hospital discharge and ensuring that postpartum complications are identified and managed before they escalate.

## Notes

Supplement 1. eFigure 1. Sample Selection Diagram eFigure 2. Childbirth Care Locations and Severe Maternal Morbidity and Mortality by Residence Location eTable 1. Rates and Timing of Severe Maternal Morbidity and Mortality Among Study Sample in South Carolina eTable 2. Full Model Results of Hazard Ratios of Severe Maternal Morbidity and Mortality eTable 3. Rates and Hazard Ratios of Severe Maternal Morbidity and Mortality in Relation to Residence and Birthing Hospital Location by Postpartum COVID-19 Exposure eTable 4. Sensitivity Analysis: Associations of Prenatal Care Continuity and Other Maternal and Hospital Characteristics With Severe Maternal Morbidity and Mortality eTable 5. Sensitivity Analysis: Full Model With Racial and Ethnic Differences in the Association Between Residence and Birthing Hospital Location and Severe Maternal Morbidity and/or Mortality

Supplement 2. Data Sharing Statement
