# Supplementary material for: Severe Maternal Morbidity and Mortality After Delivery Hospitalization Among Rural Residents Bypassing Local Care for Urban Hospitals
Source: JAMA Netw Open. 2025 Nov 19;8(11):e2544522. doi: 10.1001/jamanetworkopen.2025.44522 (PMC12631495; doi:10.1001/jamanetworkopen.2025.44522)
Supplement: Supplement 1. — eFigure 1. Sample Selection Diagram eFigure 2. Childbirth Care Locations and Severe Maternal Morbidity and Mortality by Residence Location eTable 1. Rates and Timing of Severe Maternal Morbidity and Mortality Among Study Sample in South Carolina eTable 2. Full Model Results of Hazard Ratios of Severe Maternal Morbidity and Mortality eTable 3. Rates and Hazard Ratios of Severe Maternal Morbidity and Mortality in Relation to Residence and Birthing Hospital Location by Postpartum COVID-19 Exposure eTable 4. Sensitivity Analysis: Associations of Prenatal Care Continuity and Other Maternal and Hospital Characteristics With Severe Maternal Morbidity and Mortality eTable 5. Sensitivity Analysis: Full Model With Racial and Ethnic Differences in the Association Between Residence and Birthing Hospital Location and Severe Maternal Morbidity and/or Mortality [file jamanetwopen-e2544522-s001.pdf]

Supplemental Online Content

Hung P, Gao H, Liu J, et al. Severe maternal morbidity and mortality after delivery hospitalization among rural residents bypassing local care for urban hospitals. *JAMA Netw. Open*. 2025;8(11): e2544522. doi:10.1001/jamanetworkopen.2025.44522

eFigure 1. Sample Selection Diagram

eFigure 2. Childbirth Care Locations and Severe Maternal Morbidity and Mortality by Residence Location

eTable 1. Rates and Timing of Severe Maternal Morbidity and Mortality among Study Sample in South Carolina

eTable 2. Full Model Results of Hazard Ratios of Severe Maternal Morbidity and Mortality

eTable 3. Rates and Hazard Ratios of Severe Maternal Morbidity and Mortality in Relation to Residence and Birthing Hospital Location by Postpartum COVID-19 Exposure

eTable 4. Sensitivity Analysis: Associations of Prenatal Care Continuity and other Maternal and Hospital Characteristics with Severe Maternal Morbidity and Mortality

eTable 5. Sensitivity Analysis: Full Model with Racial and Ethnic Differences in the Association between Residence and Birthing Hospital Location and Severe Maternal Morbidity and/or Mortality

This supplemental material has been provided by the authors to give readers additional information about their work.

© 2025 Hung P et al. *JAMA Network Open*.

eFigure 1. Sample Selection Diagram

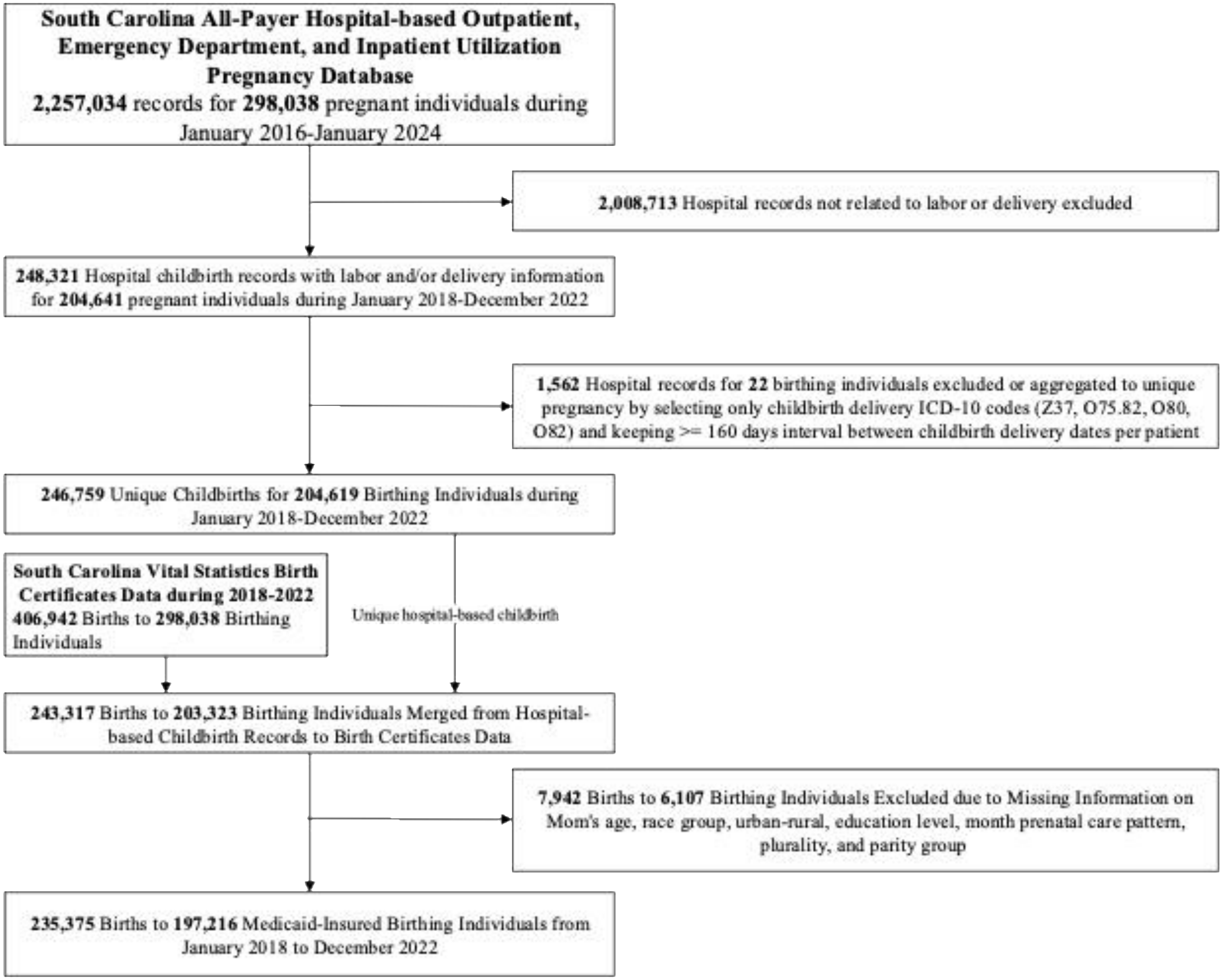

© 2025 Hung P et al. *JAMA Network Open*.

eFigure 2. Childbirth Care Locations and Severe Maternal Morbidity and Mortality by Residence Location

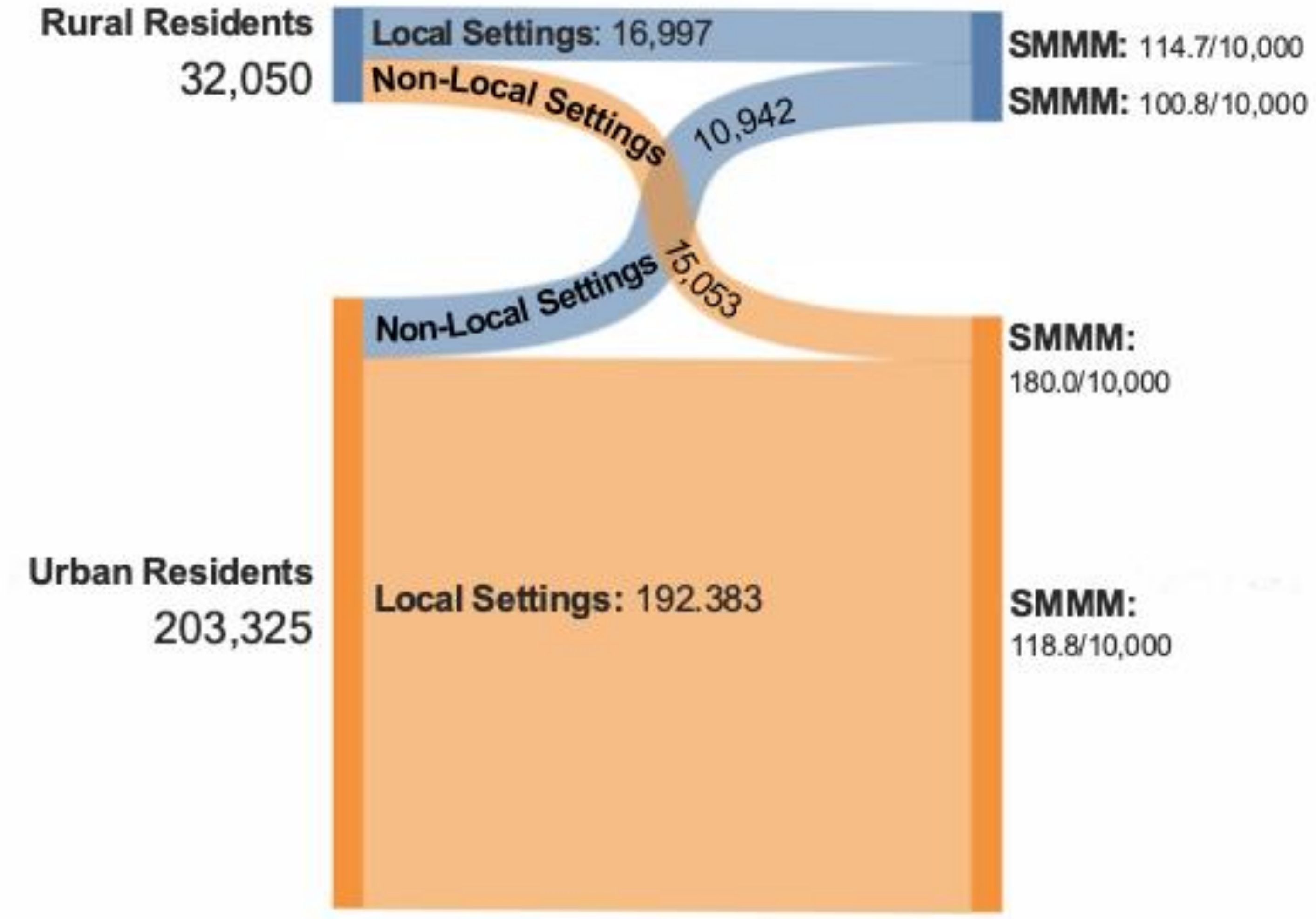

Notes: Urban/rural location, based on residence county, was defined using the 2023 Rural-Urban Continuum Codes developed by the US Department of Agriculture. Non-local settings were defined as rural residents giving birth in urban hospitals and urban residents giving birth in rural hospitals. Local settings referred as rural residents giving birth in rural hospitals and urban residents giving birth in urban hospitals.

© 2025 Hung P et al. *JAMA Network Open*.

eTable 1. Rates and Timing of Severe Maternal Morbidity and Mortality among Study Sample in South Carolina

	Severe Maternal Morbidity and Mortality without Blood Transfusion-Only			
	Number of Births with SMMM (Rate per 10,000)	P value	Median (Interquartile) of Days to First SMMM Event among SMMM-Affected Births	P values
**Study Sample**	**2,881 (122.4)**		**66 (10 - 208)**	
**Residence and Birthing Hospital Location**		<.001		<.001
Urban Residents	2,415 (118.8)		67 (10-216)	
Rural Non-Local Deliveries	271 (180.0)		67 (11-188)	
Rural Local Deliveries	195 (114.7)		59 (8-180)	
**Maternal Age at Birth**		<.001		<.001
<20	157 (123.0)		106 (14-249)	
20-24	613 (119.5)		81 (11-222)	
25-29	805 (112.4)		67 (10-205)	
30-34	766 (120.6)		64 (9-209)	
35+	540 (149.1)		48 (9-183)	
**Maternal Race and Ethnicity**		<.001		<.001
Hispanic	110 (85.9)		113 (17-254)	
Non-Hispanic Black	1,424 (199.2)		57 (9-194)	
Non-Hispanic Other[a]	123 (76.1)		48 (10-174)	
Non-Hispanic White	1,224 (90.7)		83 (11-218)	
**Maternal Education Attainment**		<.001		<.001
No high school diploma	484 (168.4)		122 (19-254)	
High school diploma	889 (145.0)		65 (11-196)	
Some college	1,051 (137.1)		71 (10-211)	
Bachelor's degree	276 (63.0)		30 (7-154)	
Graduate school	181 (72.9)		17 (7-132)	
**Pre-pregnancy BMI**		<.001		<.001
Underweight	106 (117.5)		106 (17-204)	
Healthy weight	729 (86.8)		89 (13-230)	
Overweight	648 (108.5)		67 (10-193)	
Obesity	1,398 (169.1)		56 (9-199)	
**Prenatal Care Initiation**		0.018		0.020
First Trimester	2,113 (118.8)		64 (9-203)	
Second Trimester	594 (134.5)		67 (14-205)	
Third Trimester	174 (130.5)		93 (10-244)	
**Plurality**		<.001		<.001
Singleton	2,799 (121.0)		70 (10-209)	
Multiple Birth	82 (200.9)		22 (9-192)	
**Parity**		<.001		<.001
First Birth	923 (105.3)		44 (8-193)	
Second Birth	820 (109.8)		79 (10-214)	
Third Birth	586 (137.9)		72 (12-213)	
Fourth or More Births	552 (180.5)		85 (15-216)	
**Gestational Age at Birth**		<.001		<.001
<=31 weeks	162 (460.1)		73.5 (13-212)	
32-33 weeks	104 (368.1)		123 (27-234)	
34-36 weeks	472 (256.9)		109 (14-244.5)	
37-38 weeks	1,011 (142.6)		64 (9-198)	
39-40 weeks	1,083 (81.4)		51 (9-198)	
41+ weeks	49 (79.1)		42 (7-193)	
**Mode of Delivery**		<.001		<.001

© 2025 Hung P et al. *JAMA Network Open*.

Vaginal Delivery	1,476 (94.0)	73 (10-205)
Cesarean Delivery	1,405 (179.2)	62 (10-212)
**Hospital % Childbirth Covered by Medicaid**	<.001	<.001
Quartile I: <47%	844 (100.2)	57 (9-199)
Quartile II: 47-60%	987 (124.2)	72 (10-207)
Quartile III: 61-77%	872 (148.5)	67 (11-205)
Quartile IV: 78+%	178 (137.6)	99 (11-233)
**Kotelchuck Index**	<.001	<.001
Inadequate	579 (146.8)	96 (14-231)
Intermediate	160 (129.5)	53 (10-195)
Adequate	578 (85.8)	62 (9-195)
Adequate Plus	1,555 (134.0)	59 (9-199)
**Type I, Type II, Gestational Diabetes**	<.001	<.001
No	2,303 (111.7)	62 (10-205)
Yes	578 (197.5)	88 (11-219)
**Preexisting/Pregnancy-Inducted Hypertension**	<.001	<.001
No	1,769 (97.2)	76 (11-215)
Yes	1,112 (208.2)	54 (9-196)
**Mental Health Disorder**	<.001	<.001
No	1,850 (111.5)	52 (9-198)
Yes	1,031 (148.4)	91 (15-224)
**Substance Use Disorder**	<.001	<.001
No	2,250 (116.4)	57 (9-197)
Yes	631 (149.9)	101 (21-233)
**Obstetric Comorbidity Index**	<.001	<.001
None	373 (60.3)	65 (9-210)
1-8	681 (101.4)	44 (8-193)
9-14	646 (115.7)	61 (9-208)
15+	1,181 (233.7)	86 (13-216)
**Hospital Obstetric Care Level**	<.001	<.001
I	297 (92.1)	58 (7-218)
II	895 (98.4)	56 (9-214)
III-IV	1,449 (160.7)	78 (12-204)
Missing	240 (109.3)	52 (9-193)
**Hospital Obstetric Care Workforce Model**	<.001	<.001
No Obstetrician	2 (512.8)	230 (118-342)
Obstetrician only	746 (90.8)	53 (8-204)
Both Obstetrician and Family Physician	2,133 (139.2)	74 (10-208)
**Hospital Annual Births**	<.001	<.001
<250	45 (129.2)	114 (13-253)
251-650	222 (107.7)	68 (7-222)
651-1500	686 (104.8)	56 (9-205)
1501-4337	1,928 (132.2)	71 (11-205)
**Birth Year**	0.022	0.019
2018	482 (112.3)	59 (9-212)
2019	532 (119.1)	50 (8-195)
2020	545 (117.9)	67 (11-217)
2021	660 (134.6)	78 (12-210)
2022	662 (126.1)	76 (11-208)

P values for comparisons of severe maternal morbidity and mortality rates between groups were calculated using Pearson χ^2 tests or Fisher exact tests. Log-rank tests were used to compare medians and interquartile ranges. a. Non-Hispanic Other group includes Asian, American Indian/Alaska Native, multiracial, Native Hawaiian, or Other Pacific Islander.

© 2025 Hung P et al. *JAMA Network Open*.

eTable 2. Adjusted Risk of Severe Maternal Morbidity and/or Mortality[a]

	Hazard Ratios (95% CI)		P value
	Crude	Adjusted	
**Residence and Birthing Hospital Location**			
Urban Residents	1 [Reference]	1 [Reference]	
Rural Resident Bypassed for Urban Hospitals	1.51 (1.33-1.71)	1.18 (1.04-1.33)	0.012
Rural Residents Stayed Local for Childbirth	0.97 (0.84-1.12)	0.88 (0.76-1.03)	0.109
**Maternal Age at Birth**			
<20	1.08 (0.91-1.29)	0.84 (0.7-1.01)	0.066
20-24	1.06 (0.96-1.18)	0.92 (0.82-1.02)	0.12
25-29	1 [Reference]	1 [Reference]	
30-34	1.07 (0.97-1.18)	1.21 (1.09-1.33)	<.0001
35+	1.33 (1.19-1.48)	1.41 (1.26-1.58)	<.0001
**Maternal Race and Ethnicity**			
Hispanic	0.95 (0.78-1.15)	0.74 (0.61-0.91)	0.004
Non-Hispanic Black	2.21 (2.04-2.38)	1.67 (1.54-1.81)	<.0001
Non-Hispanic Other[b]	0.84 (0.7-1.01)	0.76 (0.63-0.92)	0.004
Non-Hispanic White	1 [Reference]	1 [Reference]	
**Maternal Education Attainment**			
No high school diploma	1.16 (1.04-1.3)	1.27 (1.14-1.43)	<.0001
High school diploma	0.95 (0.87-1.04)	0.96 (0.87-1.05)	0.351
Some college	1 [Reference]	1 [Reference]	
Bachelor's degree	0.43 (0.38-0.5)	0.54 (0.47-0.62)	<.0001
Graduate school	0.5 (0.43-0.59)	0.59 (0.5-0.7)	<.0001
**Parity**			
First Birth	1 [Reference]	1 [Reference]	
Second Birth	1.04 (0.95-1.14)	0.92 (0.83-1.01)	0.084
Third Birth	1.31 (1.18-1.45)	0.97 (0.87-1.09)	0.653
Four or More Birth	1.72 (1.55-1.91)	0.96 (0.85-1.09)	0.531
**Gestational Age at Birth**			
<=31 weeks	1 [Reference]	1 [Reference]	
32-33 weeks	0.78 (0.61-1.00)	0.87 (0.68-1.12)	0.289
34-36 weeks	0.55 (0.46-0.66)	0.69 (0.58-0.83)	<.0001
37-38 weeks	0.3 (0.26-0.36)	0.49 (0.41-0.58)	<.0001
39-40 weeks	0.17 (0.15-0.21)	0.34 (0.28-0.41)	<.0001
41+ weeks	0.17 (0.12-0.23)	0.38 (0.27-0.52)	<.0001
**Kotelchuck Index**			
Inadequate	4.05 (2.07-7.93)	2.1 (1.07-4.13)	0.031
Intermediate	1 [Reference]	1 [Reference]	
Adequate	0.66 (0.56-0.79)	0.81 (0.68-0.97)	0.022
Adequate Plus	1.03 (0.88-1.22)	0.90 (0.77-1.07)	0.234
**Obstetric Comorbidity Index**			
None	1 [Reference]	1 [Reference]	
1-8	1.68 (1.48-1.91)	1.46 (1.29-1.67)	<.0001

© 2025 Hung P et al. *JAMA Network Open*.

9-14	1.93 (1.69-2.19)	1.45 (1.28-1.66)	<.0001
15+	3.9 (3.47-4.38)	1.98 (1.73-2.25)	<.0001
**Hospital Obstetric Care Level**			
I	1 [Reference]	1 [Reference]	
II	0.57 (0.51-0.65)	0.87 (0.76-1.01)	0.059
III-IV	0.61 (0.56-0.67)	0.79 (0.72-0.87)	<.0001
Missing	0.68 (0.59-0.78)	0.88 (0.76-1.01)	0.068
**Hospital Obstetric Care Workforce Model**			
No Obstetrician	5.68 (1.42-22.77)	3.86 (0.96-15.58)	0.058
Obstetrician only	1 [Reference]	1 [Reference]	
Both Obstetrician and Family Physician	1.53 (1.41-1.67)	1.12 (1.01-1.23)	0.030
**Birth Year**			
2018	1 [Reference]	1 [Reference]	
2019	1.06 (0.94-1.2)	1.02 (0.9-1.16)	0.733
2020	1.05 (0.93-1.19)	0.99 (0.87-1.12)	0.838
2021	1.2 (1.07-1.35)	1.12 (1-1.26)	0.053
2022	1.13 (1-1.27)	1.01 (0.89-1.13)	0.927

Notes: CI: Confidence Interval. a.Since blood transfusion alone may not represent a truly severe maternal event, we did not consider such cases as SMM. b. Non-Hispanic Other group includes Asian, American Indian/Alaska Native, multiracial, Native Hawaiian, or Other Pacific Islander.

© 2025 Hung P et al. *JAMA Network Open*.

eTable 3. Rates and Risk of Severe Maternal Morbidity and/or Mortality in Relation to Residence and Birthing Hospital Location by Postpartum COVID-19 Exposure

	Pre-COVID (Discharge Prior to March 2019)[a]				During COVID (Discharge after March 2020)[b]			
Characteristic	Number (Rate Per 10,000) of Severe Maternal Morbidity and/or Mortality Cases	Hazard Ratio (95% CI)		P value	Number (Rate Per 10,000) of Severe Maternal Morbidity and/or Mortality Cases	Hazard Ratio (95% CI)		P value
		Unadjusted	Adjusted[c]			Unadjusted	Adjusted[c]	
**Severe Maternal Morbidity and Mortality[d], All**	626 (113.53)				2255 (125.11)			
**Residence and Birthing Hospital Location**								
Urban Residents	523 (**110.36**)	1 [Reference]	1 [Reference]		1,892 (**121.33**)	1 [Reference]	1 [Reference]	
Rural Resident Bypassed for Urban Hospitals	48 (**142.43**)	1.30 (0.96-1.74)	0.99 (0.73-1.33)	0.924	223 (**190.88**)	1.57 (1.36-1.80)	**1.22 (1.06-1.41)**	**0.005**
Rural Residents Stayed Local for Childbirth	55 (**125.66**)	1.14 (0.87-1.51)	1.05 (0.78-1.41)	0.754	140 (**110.94**)	0.91 (0.77-1.09)	0.83 (0.70-1.06)	0.057

Notes: CI: Confidence Interval. Significant results at $p<0.05$ are **bold**.

a. Individuals never exposed to COVID-19 public health emergency gave birth prior to March 2019, resulting in a one-year postpartum period ending prior to March 2020.

b. Individuals who gave birth from March 1, 2020 to September 30, 2022, resulting in a postpartum period that fully exposed with the public health emergency

c. Adjusted hazard ratios were calculated from a multivariable cox proportional hazard model, controlling for maternal age at birth, race and ethnicity, education attainment, parity, Kotelchuck prenatal care adequacy index, gestational age at birth, obstetric comorbidity index, hospital obstetric care level, hospital obstetric care workforce model, and birth year.

d. Since blood transfusion alone may not represent a truly severe maternal event, we did not consider such cases as SMM.

© 2025 Hung P et al. *JAMA Network Open*.

eTable 4. Associations of Prenatal Care Continuity and other Maternal and Hospital Characteristics with Severe Maternal Morbidity and Mortality[a]

	Adjusted Hazard Ratio (95% CI)	P value
**Prenatal Care Location**		
No prenatal care	1 [Reference]	
Receiving prenatal care from different providers than the childbirth discharge hospital	1.00 (0.59-1.69)	0.991
Receiving prenatal care from the childbirth discharge hospital	0.72 (0.42-1.22)	0.219
**Residence and Birthing Hospital Location**		
Urban Residents	1 [Reference]	
Rural Resident Bypassed for Urban Hospitals	1.15 (1.01-1.3)	0.041
Rural Residents Stayed Local for Childbirth	0.86 (0.72-1.02)	0.082
**Maternal Age at Birth**		
<20	0.94 (0.78-1.14)	0.531
20-24	0.99 (0.89-1.11)	0.916
25-29	1 [Reference]	
30-34	1.12 (1.02-1.24)	0.024
35+	1.21 (1.08-1.36)	0.001
**Maternal Race and Ethnicity**		
Hispanic	0.65 (0.53-0.8)	<.001
Non-Hispanic Black	1.65 (1.52-1.8)	<.001
Non-Hispanic Other[b]	0.72 (0.6-0.87)	<.001
Non-Hispanic White	1 [Reference]	
**Maternal Education Attainment**		
No high school diploma	1.31 (1.17-1.47)	<.001
High school diploma	0.92 (0.84-1.01)	0.083
Some college	1 [Reference]	
Bachelor's degree	0.52 (0.45-0.6)	<.001
Graduate school	0.58 (0.49-0.69)	<.001
**Parity**		
First Birth	1 [Reference]	
Second Birth	1.00 (0.91-1.11)	0.992
Third Birth	1.11 (0.99-1.25)	0.062
Four or More Birth	1.20 (1.06-1.36)	0.005
**Gestational Age at Birth**		
<=31 weeks	1 [Reference]	
32-33 weeks	0.87 (0.68-1.12)	0.290
34-36 weeks	0.69 (0.59-0.83)	<.0001
37-38 weeks	0.49 (0.41-0.58)	<.0001
39-40 weeks	0.34 (0.28-0.42)	<.0001
41+ weeks	0.38 (0.27-0.52)	<.0001
**Kotelchuck Index**		
Inadequate	1.04 (0.86-1.27)	0.675
Intermediate	1 [Reference]	
Adequate	0.80 (0.67-0.96)	0.015
Adequate Plus	0.92 (0.78-1.09)	0.339
**Obstetric Comorbidity Index**		
None	1 [Reference]	
1-8	1.27 (1.11-1.45)	<.001

© 2025 Hung P et al. *JAMA Network Open*.

9-14	1.33 (1.16-1.52)	<.001
15+	1.71 (1.47-1.98)	<.001
**Hospital Obstetric Care Level**		
I	1 [Reference]	
II	0.84 (0.70-1.01)	0.06
III-IV	0.78 (0.69-0.89)	<.001
Missing	0.91 (0.78-1.06)	0.23
**Hospital Obstetric Care Workforce Model**		
No Obstetrician	4.26 (1.01-17.93)	0.048
Obstetrician only	1 [Reference]	
Both Obstetrician and Family Physician	1.15 (1.04-1.28)	0.008
**Birth Year**		
2018	1 [Reference]	
2019	1.02 (0.90-1.16)	0.716
2020	0.97 (0.86-1.1)	0.646
2021	1.13 (1.00-1.28)	0.052
2022	0.99 (0.87-1.13)	0.902

Notes: CI: Confidence Interval. a.Since blood transfusion alone may not represent a truly severe maternal event, we did not consider such cases as SMM. b. Non-Hispanic Other group includes Asian, American Indian/Alaska Native, multiracial, Native Hawaiian, or Other Pacific Islander.

© 2025 Hung P et al. *JAMA Network Open*.

eTable 5. Sensitivity Analysis: Full Model with Racial and Ethnic Differences in the Association between Residence and Birthing Hospital Location and Severe Maternal Morbidity and/or Mortality

	Adjusted Hazard Ratio (95% Confidence Interval)	P value
**Residence and Birthing Hospital Location**		
Urban Residents	1 [Reference]	
Rural Resident Bypassed for Urban Hospitals	1.13 (0.92-1.38)	0.259
Rural Residents Stayed Local for Childbirth	1.09 (0.86-1.38)	0.473
**Maternal Race and Ethnicity**		
Hispanic	0.75 (0.61-0.93)	0.008
Non-Hispanic Black	1.70 (1.55-1.85)	<.001
Non-Hispanic Other[a]	0.77 (0.63-0.93)	0.008
Non-Hispanic White	1 [Reference]	
**Interactions of Residence/Birthing Hospital Location and Race/Ethnicity**		
Non-Hispanic White, Urban Residents	1 [Reference]	
Non-Hispanic Black, Rural Resident Bypassed for Urban Hospitals	1.1 (0.84-1.43)	0.485
Non-Hispanic Black, Rural Residents Stayed Local for Childbirth	0.93 (0.83-1.04)	0.176
Hispanic, Rural Resident Bypassed for Urban Hospitals	1.00 (<0.01-2.06)	0.965
Hispanic, Rural Residents Stayed Local for Childbirth	1.04 (0.49-2.22)	0.917
Non-Hispanic Other, Rural Resident Bypassed for Urban Hospitals	0.88 (0.38-2.05)	0.765
Non-Hispanic Other, Rural Residents Stayed Local for Childbirth	0.97 (0.39-2.46)	0.956
**Maternal Age at Birth**		
<20	0.84 (0.7-1.01)	0.067
20-24	0.92 (0.82-1.02)	0.119
25-29	1 [Reference]	
30-34	1.21 (1.09-1.34)	<.001
35+	1.41 (1.26-1.58)	<.001
**Maternal Education Attainment**		
No high school diploma	1.27 (1.13-1.43)	<.001
High school diploma	1 [Reference]	
Some college	0.96 (0.87-1.05)	0.337
Bachelor's degree	0.54 (0.47-0.62)	<.001
Graduate school	0.59 (0.5-0.7)	<.001
**Gestational Age at Birth**		
<=31 weeks	1 [Reference]	
32-33 weeks	0.87 (0.68-1.12)	0.282
34-36 weeks	0.69 (0.58-0.83)	<.001
37-38 weeks	0.49 (0.41-0.58)	<.001
39-40 weeks	0.34 (0.28-0.41)	<.001
41+ weeks	0.38 (0.27-0.52)	<.001

© 2025 Hung P et al. *JAMA Network Open*.

**Parity**		
First Birth	1 [Reference]	
Second Birth	0.92 (0.83-1.01)	0.084
Third Birth	0.98 (0.87-1.09)	0.66
Four or More Birth	0.96 (0.85-1.09)	0.538
**Kotelchuck Index**		
Inadequate	0.95 (0.8-1.13)	0.571
Intermediate	1 [Reference]	
Adequate	0.81 (0.68-0.97)	0.022
Adequate Plus	0.9 (0.77-1.07)	0.233
**Obstetric Comorbidity Index**		
None	1.46 (1.29-1.66)	<.001
1-8	1.45 (1.28-1.66)	<.001
9-14	1.97 (1.73-2.25)	<.001
15+	1 [Reference]	
**Hospital Obstetric Care Level**	1 [Reference]	
I	0.87 (0.76-1.00)	0.052
II	0.80 (0.72-0.88)	<.001
III-IV	1 [Reference]	
Missing	0.88 (0.76-1.01)	0.074
**Hospital Obstetric Care Workforce Model**		
No OB	4.01 (0.99-16.2)	0.051
OB only	1 [Reference]	
Both OB and FP	1.11 (1.01-1.23)	0.038
**Birth Year**		
2018	1 [Reference]	
2019	1.02 (0.90-1.16)	0.734
2020	0.99 (0.87-1.12)	0.833
2021	1.12 (1.00-1.26)	0.054
2022	1.01 (0.89-1.14)	0.92

Notes: a. Non-Hispanic Other group includes Asian, American Indian/Alaska Native, multiracial, Native Hawaiian, or Other Pacific Islander.

© 2025 Hung P et al. *JAMA Network Open*.
