# Supplementary material for: Severe Maternal Morbidity and Mortality After Delivery Hospitalization Among Rural Residents Bypassing Local Care for Urban Hospitals
Source: JAMA Netw Open. 2025 Nov 19;8(11):e2544522. doi: 10.1001/jamanetworkopen.2025.44522 (PMC12631495; doi:10.1001/jamanetworkopen.2025.44522)
Supplement: Supplement 2. — Data Sharing Statement [file jamanetwopen-e2544522-s002.pdf]

Data Sharing Statement

Hung. Severe Maternal Morbidity and Mortality After Delivery Hospitalization Among Rural Residents Bypassing Local Care for Urban Hospitals. *JAMA Netw Open*. Published November 19, 2025. doi:10.1001/jamanetworkopen.2025.44522

Data
Data available: No

Additional Information
Explanation for why data not available: The data used in this study are not publicly available but can be accessed through the South Carolina Revenue and Fiscal Affairs Office (RFA) upon request. Researchers may apply for data access following RFA's established protocols and data use agreements. Requests for access must comply with institutional and state regulations governing the use of protected health information.
